# *rac*-(*S*)-2-(1*H*-Imidazol-1-yl)-3-methyl­butan-1-ol

**DOI:** 10.1107/S1600536809004565

**Published:** 2009-02-13

**Authors:** Guangfu Song, Fang Xue, Dongliang Li

**Affiliations:** a Technical Research Center, China Tabacco Chuanyu Industrial Corporation, Chengdu 610066, People’s Republic of China

## Abstract

In the crystal structure of the title compound, C_8_H_14_N_2_O, inter­molecular O—H⋯N hydrogen bonds link mol­ecules related by translation along the *a* axis into chains. Weak inter­molecular C—H⋯O hydrogen bonds and C—H⋯π inter­actions enhance the crystal packing stability.

## Related literature

For useful applications of imidazole derivatives, see Lu *et al.* (2006 ▶); Zou *et al.* (2006 ▶). For details of the synthesis, see Bao *et al.* (2003 ▶); Guo *et al.* (2006 ▶).
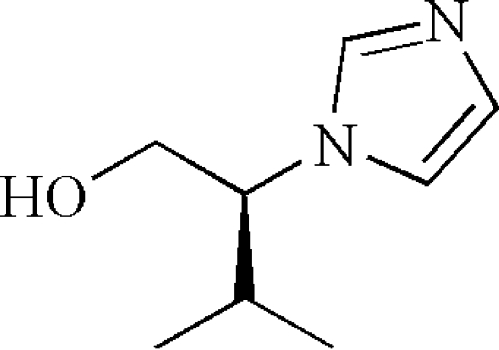


## Experimental

### 

#### Crystal data

C_8_H_14_N_2_O
*M* *_r_* = 154.21
Monoclinic, 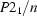

*a* = 7.356 (4) Å
*b* = 7.212 (3) Å
*c* = 16.549 (5) Å
β = 90.54 (3)°
*V* = 877.9 (7) Å^3^
*Z* = 4
Mo *K*α radiation
μ = 0.08 mm^−1^
*T* = 292 K
0.58 × 0.54 × 0.42 mm

#### Data collection

Enraf–Nonius CAD-4 diffractometer
Absorption correction: none
1931 measured reflections
1630 independent reflections
965 reflections with *I* > 2σ(*I*)
*R*_int_ = 0.004
3 standard reflections every 120 reflections intensity decay: 0.3%

#### Refinement

*R*[*F* ^2^ > 2σ(*F* ^2^)] = 0.067
*wR*(*F* ^2^) = 0.197
*S* = 1.18
1630 reflections
104 parameters
H-atom parameters constrained
Δρ_max_ = 0.31 e Å^−3^
Δρ_min_ = −0.24 e Å^−3^

### 

Data collection: *DIFRAC* (Gabe & White, 1993 ▶); cell refinement: *DIFRAC*; data reduction: *NRCVAX* (Gabe *et al.*, 1989 ▶); program(s) used to solve structure: *SHELXS97* (Sheldrick, 2008 ▶); program(s) used to refine structure: *SHELXL97* (Sheldrick, 2008 ▶); molecular graphics: *ORTEPIII* (Burnett & Johnson, 1996 ▶); software used to prepare material for publication: *SHELXL97* and *PLATON* (Spek, 2009 ▶).

## Supplementary Material

Crystal structure: contains datablocks global, I. DOI: 10.1107/S1600536809004565/cv2517sup1.cif

Structure factors: contains datablocks I. DOI: 10.1107/S1600536809004565/cv2517Isup2.hkl

Additional supplementary materials:  crystallographic information; 3D view; checkCIF report

## Figures and Tables

**Table 1:** Hydrogen-bond geometry (Å, °)

*D*—H⋯*A*	*D*—H	H⋯*A*	*D*⋯*A*	*D*—H⋯*A*
O1—H1⋯N2^i^	0.82	1.93	2.751 (3)	176
C1—H1*A*⋯O1^ii^	0.93	2.43	3.353 (4)	173
C4—H4⋯*Cg*^ii^	0.98	2.86	3.716 (4)	146

Symmetry codes: (i) 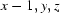; (ii) 
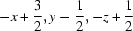
. *Cg* is the centroid of the C1–C3/N1/N2  ring.

**Fig. 1.**
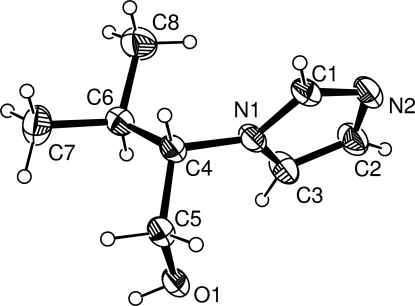
The molecular structure of (I), showing 30% probability displacement ellipsoids and the atomic numbering.

## supplementary crystallographic information

### Comment

Imidazole is important for biological systems, and its derivatives have attracted widespread interest due to their further expanded application in perfume chemistry and in the construction of some interesting metal–organic frameworks (Lu *et al.* 2006; Zou *et al.* 2006). Here, we report the crystal structure of the title compound, (I), which is a basic unit of constructing chiral receptors and could be applied for the preparation of perfume.

As shown in Fig. 1, there is a chiral center at C4 derived from the source *L*-valine. In the crystal, intermolecular O—H···N hydrogen bonds (Table 1) link the molecules related by translation along axis *a* into chains. Weak intermolecular C—H···O hydrogen bonds and C—H···π interactions (Table 1) enhance the crystal packing stability.

### Experimental

The title compound was prepared according to the literature (Guo *et al.* 2006). Starting from *L*-valine, methyl 2-(1*H*-imidazol-1-yl)-3-methylbutanoate was easily prepared according to literature procedure (Bao *et al.* 2003). Following, NaBH_4_ (1.52 g, 40 mmol) was added to methyl 2-(1*H*-imidazol-1-yl)-3-methylbutanoate (1.82 g, 10.0 mmol) in ethanol (50 ml) at 273 K during 30 min. The mixture was stirred at 333 K for another 20 h and then evaporated under vacuum. The residue was diluted with 50 ml saturated K_2_CO_3_ and extracted with 30 ml ethyl acetate. The organic layer was dried over anhydrous Na_2_SO_4_ and concentrated under reduced pressure. The resulting residue was purified by column chromatography on silica gel eluting with CH_2_Cl_2_/CH_3_OH (20/1, v/v). Then, colourless crystals suitable for X-ray analysis can be obtained by recrystallization of the compound from ethyl acetate.

### Refinement

All H atoms were positioned geometrically and refined in the riding model approximation, with C—H = 0.93–0.98 Å and O—H = 0.82 Å, and U_iso_(H) = 1.2–1.5 U_eq_ of the parent atom.

### Crystal data

**Table:**

C_8_H_14_N_2_O	*F*(000) = 336
*M**_r_* = 154.21	*D*_x_ = 1.167 Mg m^−^^3^
Monoclinic, *P*2_1_/*n*	Mo *K*α radiation, λ = 0.71073 Å
Hall symbol: -P 2yn	Cell parameters from 21 reflections
*a* = 7.356 (4) Å	θ = 4.6–7.4°
*b* = 7.212 (3) Å	µ = 0.08 mm^−^^1^
*c* = 16.549 (5) Å	*T* = 292 K
β = 90.54 (3)°	Block, colourless
*V* = 877.9 (7) Å^3^	0.58 × 0.54 × 0.42 mm
*Z* = 4	

### Data collection

**Table:**

Enraf–Nonius CAD-4 diffractometer	*R*_int_ = 0.004
Radiation source: fine-focus sealed tube	θ_max_ = 25.5°, θ_min_ = 2.5°
graphite	*h* = −8→8
ω/2θ scans	*k* = 0→8
1931 measured reflections	*l* = −7→20
1630 independent reflections	3 standard reflections every 120 reflections
965 reflections with *I* > 2σ(*I*)	intensity decay: 0.3%

### Refinement

**Table:**

Refinement on *F*^2^	Secondary atom site location: difference Fourier map
Least-squares matrix: full	Hydrogen site location: inferred from neighbouring sites
*R*[*F*^2^ > 2σ(*F*^2^)] = 0.067	H-atom parameters constrained
*wR*(*F*^2^) = 0.197	*w* = 1/[σ^2^(*F*_o_^2^) + (0.0941*P*)^2^] where *P* = (*F*_o_^2^ + 2*F*_c_^2^)/3
*S* = 1.18	(Δ/σ)_max_ < 0.001
1630 reflections	Δρ_max_ = 0.31 e Å^−^^3^
104 parameters	Δρ_min_ = −0.24 e Å^−^^3^
0 restraints	Extinction correction: *SHELXS97* (Sheldrick, 2008), Fc^*^=kFc[1+0.001xFc^2^λ^3^/sin(2θ)]^-1/4^
Primary atom site location: structure-invariant direct methods	Extinction coefficient: 0.046 (11)

### Special details

**Table:**

Geometry. All e.s.d.'s (except the e.s.d. in the dihedral angle between two l.s. planes) are estimated using the full covariance matrix. The cell e.s.d.'s are taken into account individually in the estimation of e.s.d.'s in distances, angles and torsion angles; correlations between e.s.d.'s in cell parameters are only used when they are defined by crystal symmetry. An approximate (isotropic) treatment of cell e.s.d.'s is used for estimating e.s.d.'s involving l.s. planes.
Refinement. Refinement of *F*^2^ against ALL reflections. The weighted *R*-factor *wR* and goodness of fit *S* are based on *F*^2^, conventional *R*-factors *R* are based on *F*, with *F* set to zero for negative *F*^2^. The threshold expression of *F*^2^ > σ(*F*^2^) is used only for calculating *R*-factors(gt) *etc*. and is not relevant to the choice of reflections for refinement. *R*-factors based on *F*^2^ are statistically about twice as large as those based on *F*, and *R*- factors based on ALL data will be even larger.

### Fractional atomic coordinates and isotropic or equivalent isotropic displacement parameters (Å^2^)

**Table:**

	*x*	*y*	*z*	*U*_iso_*/*U*_eq_	
O1	0.4065 (2)	0.1870 (3)	0.31165 (14)	0.0557 (7)	
H1	0.3011	0.1697	0.3255	0.084*	
N1	0.7629 (3)	0.0340 (3)	0.34211 (14)	0.0459 (7)	
N2	1.0479 (3)	0.1263 (4)	0.34916 (16)	0.0598 (8)	
C4	0.6043 (3)	−0.0816 (4)	0.32307 (17)	0.0456 (8)	
H4	0.6473	−0.1848	0.2898	0.055*	
C5	0.4713 (4)	0.0284 (4)	0.27157 (18)	0.0480 (8)	
H5A	0.5306	0.0660	0.2221	0.058*	
H5B	0.3692	−0.0503	0.2570	0.058*	
C1	0.9353 (4)	0.0047 (4)	0.31778 (19)	0.0514 (8)	
H1A	0.9698	−0.0901	0.2830	0.062*	
C3	0.7676 (4)	0.1855 (4)	0.39170 (18)	0.0559 (8)	
H3	0.6696	0.2406	0.4174	0.067*	
C6	0.5261 (4)	−0.1657 (4)	0.39954 (16)	0.0487 (8)	
H6	0.4769	−0.0643	0.4322	0.058*	
C2	0.9438 (4)	0.2392 (4)	0.3958 (2)	0.0597 (9)	
H2	0.9875	0.3387	0.4260	0.072*	
C7	0.3712 (5)	−0.2990 (5)	0.3805 (2)	0.0693 (10)	
H7A	0.4164	−0.4013	0.3495	0.104*	
H7B	0.2786	−0.2355	0.3500	0.104*	
H7C	0.3208	−0.3444	0.4300	0.104*	
C8	0.6711 (5)	−0.2629 (5)	0.4495 (2)	0.0761 (11)	
H8A	0.6199	−0.3035	0.4996	0.114*	
H8B	0.7693	−0.1786	0.4602	0.114*	
H8C	0.7157	−0.3681	0.4202	0.114*	

### Atomic displacement parameters (Å^2^)

**Table:**

	*U*^11^	*U*^22^	*U*^33^	*U*^12^	*U*^13^	*U*^23^
O1	0.0302 (11)	0.0505 (12)	0.0864 (15)	0.0012 (9)	−0.0025 (10)	−0.0011 (11)
N1	0.0288 (13)	0.0493 (13)	0.0595 (15)	0.0029 (11)	−0.0026 (10)	−0.0055 (11)
N2	0.0307 (14)	0.0628 (16)	0.086 (2)	−0.0005 (12)	−0.0039 (13)	−0.0031 (14)
C4	0.0325 (15)	0.0424 (15)	0.0617 (18)	−0.0002 (12)	−0.0060 (13)	−0.0027 (14)
C5	0.0355 (16)	0.0499 (16)	0.0586 (17)	−0.0024 (13)	−0.0038 (13)	−0.0008 (14)
C1	0.0334 (16)	0.0518 (17)	0.069 (2)	0.0086 (14)	0.0035 (14)	−0.0014 (14)
C3	0.0353 (16)	0.0655 (19)	0.0669 (19)	0.0006 (15)	−0.0020 (14)	−0.0129 (16)
C6	0.0407 (16)	0.0520 (17)	0.0532 (17)	0.0002 (14)	−0.0043 (13)	0.0040 (14)
C2	0.0405 (17)	0.0628 (19)	0.075 (2)	−0.0027 (15)	−0.0125 (15)	−0.0133 (16)
C7	0.066 (2)	0.071 (2)	0.071 (2)	−0.0228 (19)	0.0010 (17)	0.0087 (18)
C8	0.063 (2)	0.085 (2)	0.081 (2)	0.0034 (19)	−0.0120 (19)	0.025 (2)

### Geometric parameters (Å, °)

**Table:**

O1—C5	1.408 (3)	C3—C2	1.354 (4)
O1—H1	0.8200	C3—H3	0.9300
N1—C1	1.351 (4)	C6—C8	1.515 (4)
N1—C3	1.367 (3)	C6—C7	1.522 (4)
N1—C4	1.466 (3)	C6—H6	0.9800
N2—C1	1.310 (3)	C2—H2	0.9300
N2—C2	1.362 (4)	C7—H7A	0.9600
C4—C5	1.516 (3)	C7—H7B	0.9600
C4—C6	1.521 (4)	C7—H7C	0.9600
C4—H4	0.9800	C8—H8A	0.9600
C5—H5A	0.9700	C8—H8B	0.9600
C5—H5B	0.9700	C8—H8C	0.9600
C1—H1A	0.9300		
			
C5—O1—H1	109.5	N1—C3—H3	126.9
C1—N1—C3	106.6 (2)	C8—C6—C4	111.6 (2)
C1—N1—C4	126.5 (2)	C8—C6—C7	110.0 (3)
C3—N1—C4	126.8 (2)	C4—C6—C7	111.6 (2)
C1—N2—C2	105.6 (2)	C8—C6—H6	107.8
N1—C4—C5	109.4 (2)	C4—C6—H6	107.8
N1—C4—C6	110.8 (2)	C7—C6—H6	107.8
C5—C4—C6	115.4 (2)	C3—C2—N2	110.1 (3)
N1—C4—H4	107.0	C3—C2—H2	125.0
C5—C4—H4	107.0	N2—C2—H2	125.0
C6—C4—H4	107.0	C6—C7—H7A	109.5
O1—C5—C4	112.3 (2)	C6—C7—H7B	109.5
O1—C5—H5A	109.1	H7A—C7—H7B	109.5
C4—C5—H5A	109.1	C6—C7—H7C	109.5
O1—C5—H5B	109.1	H7A—C7—H7C	109.5
C4—C5—H5B	109.1	H7B—C7—H7C	109.5
H5A—C5—H5B	107.9	C6—C8—H8A	109.5
N2—C1—N1	111.6 (3)	C6—C8—H8B	109.5
N2—C1—H1A	124.2	H8A—C8—H8B	109.5
N1—C1—H1A	124.2	C6—C8—H8C	109.5
C2—C3—N1	106.1 (3)	H8A—C8—H8C	109.5
C2—C3—H3	126.9	H8B—C8—H8C	109.5
			
C1—N1—C4—C5	−115.0 (3)	C1—N1—C3—C2	−0.8 (3)
C3—N1—C4—C5	69.3 (3)	C4—N1—C3—C2	175.7 (2)
C1—N1—C4—C6	116.8 (3)	N1—C4—C6—C8	−51.7 (3)
C3—N1—C4—C6	−59.0 (3)	C5—C4—C6—C8	−176.6 (2)
N1—C4—C5—O1	−61.5 (3)	N1—C4—C6—C7	−175.2 (2)
C6—C4—C5—O1	64.2 (3)	C5—C4—C6—C7	59.9 (3)
C2—N2—C1—N1	0.0 (3)	N1—C3—C2—N2	0.8 (4)
C3—N1—C1—N2	0.5 (3)	C1—N2—C2—C3	−0.5 (4)
C4—N1—C1—N2	−175.9 (2)		

### Hydrogen-bond geometry (Å, °)

**Table:**

*D*—H···*A*	*D*—H	H···*A*	*D*···*A*	*D*—H···*A*
O1—H1···N2^i^	0.82	1.93	2.751 (3)	176
C1—H1A···O1^ii^	0.93	2.43	3.353 (4)	173
C4—H4···Cg^ii^	0.98	2.86	3.716 (4)	146

Symmetry codes: (i) *x*−1, *y*, *z*; (ii) −*x*+3/2, *y*−1/2, −*z*+1/2.
